# Psychometric properties of the Farsi translation of the kiddie schedule for affective disorders and schizophrenia-present and lifetime version

**DOI:** 10.1186/1471-244X-6-10

**Published:** 2006-03-15

**Authors:** Ahmad Ghanizadeh, Mohammad Reza Mohammadi, Arash Yazdanshenas

**Affiliations:** 1 Department of Child and Adolescent Psychiatry, Shiraz University of Medical Sciences, Hafez Hospital, Shiraz, Iran; 2 Department of Child and Adolescent Psychiatry, Tehran University of Medical Sciences, Roozbeh Hospital, Tehran, Iran; 3 Shiraz University of Medical Sciences, Hafez Hospital, Shiraz, Iran

## Abstract

**Background:**

Semi-structural clinical interviews are very important in the area of mental health research and services. There were no studies of the reliability and validity of the Farsi (Persian) version of Kiddie Schedule for Affective Disorders and Schizophrenia-Present and Lifetime Version (K-SADS-PL) in Iran. This study compares the results of face-to-face, semi-structural interview and clinical interview by a child and adolescent psychiatrist.

**Method:**

Subjects were 109 children and adolescents recruited to the child and adolescent psychiatry outpatient clinic of Hafez Hospital. Order of interview (in-psychiatrist or the semi-structural interview) was determined using random assignment within a counterbalanced framework. After, translation and back translation of K-SADS-PL, the Farsi version of K-SADS-PL was provided and used in the study. The interviewer was unaware of the child and adolescent psychiatrist diagnosis at the time of making the interview. Consensual validity, test-retest and inter-rater reliability, sensitivity, specifity, positive and negative predictive validity for the disorders were studied.

**Results:**

Consensual validity of all of the psychiatric disorders was good to excellent. It was highest for panic disorder, conduct disorder, and simple phobia. Consensual validity of anorexia nervosa was 0.49. There was sufficient validity and test-retest and inter-rater reliability and good to excellent sensitivity and specifity and positive and negative predictive validity for nearly all of the disorders. Test-retest reliabilities of attention deficit hyperactivity disorder (ADHD), oppositional defiant disorder (ODD), and tic disorder were 0.81, 0.67, and 0.56; respectively. Inter-rater reliabilities of ADHD, and ODD were 0.69 and 0.69. Tic disorder, post traumatic disorder, panic disorder, and ADHD had the highest positive predictive validities.

**Conclusion:**

The Farsi version of K-SADS-PL is a valid and reliable interview instrument for use in assessing and diagnosising child and adolescent psychiatric disorders.

## Background

For comparative cross-cultural studies, adoption of diagnostic systems from other cultures should be validated to make sure validation and applicability of the instrument. This makes an objective and replicable diagnostic system. There are many diagnostic systems in other countries. Unfortunately there is not any validated and reliable diagnostic system in Iran. It is essential for successful research and lack of this instrument is a major limitation for child and adolescent psychiatry research in Iran.

Kiddie Schedule for Affective Disorders and Schizophrenia-Present and Lifetime Version (K-SADS-PL) [1] is a semi structured interview schedule for assessing psychiatric disorders in children and adolescents. At first it was a downward extension from the adults SADS [2]. The first edition was developed by Puig-Antich and was used for validation of early onset major depressive disorder [3,4]. K-SADS-PL designed for interviewing both the parents and children and has been adapted to the DSM-IV diagnostic criterion [1]. K-SADS-PL is a semi-structured interview. It has been used to assess the status of 32 DSM-IV child and adolescent psychiatric diagnosis.

It allows the interviewers to punctuate the questions by clinical judgment. Interviewers' training greatly impact on its performance. Its reliability and validity for child and adolescent psychiatric diagnosis has been reported to be sufficient [1]. It has been used in many different epidemiological, clinical and treatment of child and adolescent psychiatric studies.

Consensual validity or the degree of agreement between a semi-structured instrument and simultaneous clinical child and adolescent psychiatrist interview is one of the important aspects which determining usefulness.

Inter-rater reliability is the extent to which two raters agree on interview results.

To our knowledge, this is the first study that has surveyed psychometric properties of a semi-structural interview in child and adolescent psychiatry in Iran. K-SADS-PL is a diagnostic instrument in English, so this study was designed to develop a Farsi version. Therefore, we translated and back-translated the K-SADS-PL in Farsi and English, and its reliability and validity were studied.

## Methods

### Subjects

Data on K-SADS-PL-F consensual validity corresponds to 109 subjects, 96 outpatients child and adolescents form outpatient clinic of Hafez Hospital and 13 normal controls. They were recruited consecutively. The mean age of the subjects was 11.2 (range 4–19 years old). 55.4% were boys. The normal control group was recruited from a pediatric clinic. They were in accompanying with their parents and they had no any current or past psychiatric disorders or serious medical illness. They were referred for routine check up. All the subjects and their parents provided informed consent before participating in the study. The study was conducted according to the *Good Clinical Practice Guidelines*, in accordance with the Declaration of Helsinki, 1975, as revised in 2000; and approved by the University. Permission for the study was asked and obtained from those who developed the K-SADS-PL.

### Measure

K-SADS-PL is a semi-structured diagnostic interview for children and adolescents aged 6 to 18 years, based on DSM-IV diagnostic criteria [5]. It has essential questions for any diagnosis, which if not met, allow the interviewer to proceed to the next disorder. The symptoms are codified as present or absent. It covers all the most frequent psychiatric disorders in childhood and adolescence including of mood disorders (major depression, and mania), anxiety disorders (separation anxiety, panic disorder, agoraphobia, social phobia, simple phobia, generalized anxiety disorder, obsessive-compulsive disorder, post-traumatic stress disorder), eating disorders (anorexia and bulimia), attention-deficit/hyperactivity disorder (ADHD), conduct disorder and oppositional defiant disorder, elimination disorders (enuresis and encopresis), Tic disorder, psychotic disorders.

### Translation and back-translation of KSADS-PL

The K-SADS-PL was translated by a team of child and adolescent researchers. They had translated the K-SADS-PL into Farsi. The K-SADS-PL-Farsi Version (K-SADS-PL-F) was back-translated by a bilingual child and adolescent psychiatrist. The back-translated version of the KSADS-PL-F was reviewed and reconfirmed by the team and the final translation was fixed by consensus. To examine the feasibility of the K-SADS-PL-F it had been administered to children in a child and adolescent psychiatry clinic. After approval of its performance and establishments of face validity and content validity, it was used in the current study.

### Procedure

The demographic characteristics were asked and then the K-SADS-PL-F was administered to children and their parents by the interviewer (AY), trained in the use of the interview, diagnostic classification and making differential diagnosis. He was a physician who had passed an extensive two-month course of training about K-SADS and psychiatry. Also, in a pilot study he had interviewed with patients under supervision of the child and adolescent psychiatrist. Another interviewer was the trained child and adolescent psychiatrist (AG). The interviewer was unaware of the clinician's diagnosis at the time of making the interview.

### Consensual validity

Consensual validity or the degree of agreement of diagnosis made by the K-SADS-PL-F interview and clinical child and adolescent psychiatrist interview was studied.

### Inter-rater and test re-test reliability

The stability of the translated instrument (K-SADS-PL-F) was tested in the Iranian culture by a test-retest design on 11 subjects who were randomly selected, which all of them were from patients group. They were conducted at four-week intervals. Inter-rater reliability was tested on 14 subjects of patients group who were randomly selected. The two raters interviewed in succession, not in a conjoint session.

### Statistical analysis

The Chi-squared test was used between two interview diagnoses to determine the sensitivity, specifity, positive predictive validity, negative predictive validity, and the degree of agreement between the two interviews.

The concordance analysis of diagnoses generated by the clinician and the K-SADS-PL-F administrator was done by the kappa coefficient (κ).

The significance level (*P *value) for statistical procedures was .05.

## Results

### Diagnostic characteristic of the subjects

The frequency of psychiatric disorders made by the child and adolescent psychiatrist were; ADHD, 57.8%; oppositional defiant disorder, 50.5%; conduct disorder, 22.9%; Tic disorder, 9.2%; bipolar mood disorders, 17.4%; major depressive disorder, 6.4%; separation anxiety disorder, 19.2%; panic disorder, 1.8%; social phobia, 11.0%; simple phobia, 18.3%; generalized anxiety disorder, 17.4%; obsessive-compulsive disorder, 9.2%; post-traumatic stress disorder, 9.2%; anorexia nervosa, 0.9%; enuresis, 15.6%; encopresis, 0.9%.

The consensual validity of diagnosis by the K-SADS-PL-F is shown on Table 1. Panic disorder, conduct disorder, Simple Phobia, and ADHD had the highest kappa score for consensual validity.

**Table 1:** Consensual validity of diagnosis by the K-SADS-PL-F.

**Diagnosis**	**Number of patients**	**Kappa**	**Significance**
Attention Deficit Hyperactivity Disorder	67	0.905	0.001
	43*	0.69*	<0.001*
Oppositional Defiant Disorder	66	0.798	0.001
	4*	0.41*	<0.001*
Conduct Disorder	26	0.923	0.001
Tic Disorder	9	0.89	0.001
	17*	0.42*	<0.001*
Separation anxiety disorder	36	0.63	0.001
Panic disorder	2	1.0	0.001
Generalized anxiety disorder	27	0.781	0.001
Obsessive compulsive disorder	15	0.890	0.001
Social phobia	23	0.873	0.001
Simple phobia	10	0.913	0.001
Post traumatic disorder	8	0.879	0.001
Enuresis	21	0.873	0.001
Encopresis	2	0.662	0.001
Anorexia nervosa	3	0.492	0.001
Major depressive disorder	10	0.809	0.001
Bipolar Mood disorder	17	0.800	0.001

* Statistics from the prior study for Korean Version of the K-SADS-PL [7].

Test-retest reliability of the K-SADS-PL-F diagnosis is shown in Table 2, and Table 3 shows the inter-rater reliability of diagnosis. Sensitivity, specifity, positive predictive validity, negative predictive validity of the K-SADS-PL-F are indicated in Table 4. The highest positive predictive validity was for tic disorder, panic disorder, and post traumatic disorder. The lowest positive predictive values were for anorexia nervosa, encopresis, and separation anxiety disorder. Negative predictive validity was very high for all of the disorders.

**Table 2:** Test-retest reliability of the K-SADS-PL-F diagnosis.

**Diagnosis**	**Number of patients**	**Kappa**	**Significance**
Attention Deficit Hyperactivity Disorder	7	0.81	0.001
	10*	0.42*	0.08*
Oppositional Defiant Disorder	7	0.60	0.08
Tic disorder	4	0.56	0.3
	3*	0.42*	0.08*

* Statistics from the prior study for Korean Version of the K-SADS-PL [7].

**Table 3:** Inter-rater reliability of the K-SADS-PL-F diagnosis.

**Diagnosis**	**Number of patients**	**Kappa**	**Significance**
Attention Deficit Hyperactivity Disorder	10	0.69	0.006
Oppositional Defiant Disorder	10	0.69	0.006
Conduct disorder	4	0.81	0.002
Separation anxiety disorder	5	0.66	0.009
Enuiresis	5	0.66	0.009

**Table 4:** Sensitivity, specifity, positive predictive validity, negative predictive validity of the K-SADS-PL-F.

**Diagnosis**	**Number of patients**	**Sensitivity**	**Specifity**	**Positive predictive value**	**Negative predictive value**
Attention Deficit Hyperactivity Disorder	67	100	89.1	92.6	100
	43*	0.77*	.94*	0.95*	0.75*
Oppositional Defiant Disorder	66	100	79.6	83.3	100
	4*	0.40*	0.97*	0.50*	0.96*
Conduct Disorder	26	96	97.6	92.3	98.8
Tic Disorder	9	81.8	100	100	98
	17*	0.47*	0.92*	0.61*	0.86*
Separation anxiety disorder	36	100	82	55.6	100
Obsessive compulsive disorder	10	90	99	90	99
Panic disorder	2	100	100	100	100
Generalized anxiety disorder	27	100	91.1	70.4	100
Social phobia	15	100	96.9	80	100
Simple phobia	23	100	96.9	87	100
Post traumatic disorder	8	80	100	100	98
Enuresis	21	100	95.7	81	100
Encopresis	2	100	99.1	50	100
Anorexia nervosa	3	100	98.1	33.3	100
Major depressive disorder	10	100	97.1	70	100
Bipolar Mood disorder	17	78.9	97.8	88.2	95.8

* Statistics from the prior study for Korean Version of the K-SADS-PL [7].

## Discussion

Results of the comparison of the number of diagnoses between the K-SADS-PL Farsi version and clinical diagnosis allow us to use the K-SADS-PL-F interview with some guarantees when trying to make the psychiatric diagnosis in child and adolescents in Farsi population. There are some studies that indicated that the K-SADS-PL has sufficient validity and reliability in other countries [6,7]. A pilot inter-rater reliability study of K-SADS-P for Greek children and adolescents showed that the Kappa statistic of the diagnosis for depressive disorders was 0.90 and inter-rater reliability of assessment of anxiety symptoms was lower (*r *= 0.59) than that for depressive disorders. The Kappa for conduct disorders was also high (κ = 0.90) [8].

Some of the interviewees had difficulties in understanding some of the K-SADS-PL-F questions, especially in the depression and anxiety sections. A possible solution of these difficulties would be to more clarify the questions, to decrease the influence of clinical judgment in the questionnaire.

We should be cautious because results were achieved among a small number of raters at one site who had trained and worked together intensively. The K-SADS-PL-F administrator was a physician who had passed an extensive two-month course of training. Also, in a pilot study he had interviewed patients under the supervision of the child and adolescent psychiatrist.

Also, the socio-economic status of the subjects was not studied because there is no appropriate instrument, and many families would not like to disclose their income. Also, asking about income could have had a negative impact on their cooperation.

Data were largely collected from a clinic sample of child and adolescent psychiatry and diagnostic severity may influence the measurement of reliability. Reliability coefficients are higher in more severely affected groups [9]. Table 1 shows that no psychotic disorders or substance use disorders were diagnosed, which probably is a consequence of the sampling method. So, it is important to examine the psychometric properties of the K-SADS-PL within a large non-clinic based sample and in children with other diagnoses such as schizophrenia, before it can be recommended for widespread use in other settings.

Future investigations with the K-SADS-PL-F will examine age-, sex-, and education-specific reference values to determine its validity and reliability for current and lifetime psychiatric disorders. The current study only surveyed the current psychiatric disorders. The number of subjects for studying of test-retest and inter-rater reliability were relatively low and it should be considered in the interpretation of the results.

However, the clinical diagnosis which was made by the child and adolescent psychiatrist, diversity of the disorders, and a mixed population of child and adolescents were the strong point of this study.

## Conclusion

The Farsi version of the K-SADS-PL has sufficient consensual validity, positive and negative predictive validity and inter-rater and test re-test reliability in assessment of child and adolescent psychiatric disorders.

## Competing interests

The author(s) declare that they have no competing interests.

## Authors' contributions

AG: Assistant Professor. Conception, principal investigator, designer, statistical analysis, interpretation, psychiatrist. MRM: Professor. Conception, designer, principal investigator, member of the translation and back-translation team, psychiatrist. AY: Medical student. Investigator, statistical analysis.

All authors read and approved the final manuscript.

## Pre-publication history

The pre-publication history for this paper can be accessed here:


